# β_2_-Adrenergic receptor (β_2_-AR) agonist formoterol suppresses differentiation of L6 myogenic cells by blocking PI3K–AKT pathway

**DOI:** 10.1080/19768354.2018.1561516

**Published:** 2019-02-22

**Authors:** So-Hyeon Kim, Sun-Ju Yi, Hyerim Lee, Ji-Hyun Kim, Myung-ju Oh, Eun-Ju Song, Kyunghwan Kim, Byung H. Jhun

**Affiliations:** a School of Biological Sciences, College of Natural Sciences, Chungbuk National University, Cheongju, Chungbuk, Republic of Korea; b Department of Cogno-Mechatronics Engineering, Pusan National University, Busan, Republic of Korea

**Keywords:** L6 myoblasts, muscle differentiation, β_2_-adrenergic receptor (β_2_-AR), formoterol, AKT pathway

## Abstract

β_2_-Adrenergic receptor (β_2_-AR) is implicated in muscle metabolic activities such as glycogen metabolism, glucose uptake, lipolysis and muscle growth. However, the functional role of β_2_-AR in the differentiation of skeletal muscle is largely unknown. Here, we examined the functional role of β_2_-AR in L6 myoblast differentiation using the long-term-acting β_2_-AR-specific agonist formoterol. We observed that formoterol treatment strongly suppressed L6 myoblast differentiation and the expression of myosin heavy chain (MHC) in a dose- and time-dependent manner. Showing that both long-acting agonist (formoterol) and short-acting agonist (terbutaline) inhibited the induction of MHC protein, whereas β_2_-AR antagonist (ICI-118,551) upregulated MHC expression, we clearly demonstrated that β_2_-AR is involved in L6 myoblast differentiation. Furthermore, our pharmacological inhibition study revealed that the PI3K–AKT pathway is the main signaling pathway for myotube formation. Formoterol inhibited the activation of PI3K–AKT signaling, but not that of ERK signaling. Moreover, formoterol selectively inhibited AKT activation by IGF-I, but not by insulin. Collectively, our findings reveal a previously undocumented role of β_2_-AR activation in modulating the differentiation of L6 myoblasts.

## 1. Introduction

Skeletal muscle differentiation or myogenesis is a finely controlled process in muscle development during embryogenesis and the regeneration of skeletal muscle after injury (Tedesco et al. 2010; Chal and Pourquie 2017). During embryonic development, it begins with the determination of myogenic precursors from pluripotent mesodermal stem cells and ends with the differentiation of committed myoblasts. Myoblast differentiation includes cell cycle exit, the expression of muscle-specific proteins, and the formation and maturation of myofibers, which is predominantly regulated by myogenic regulatory factors including MyoD, Myf5, myogenin, and MRF4 (Nie et al. 2017; Cervelli et al. 2018). The transcription factors Myf5 and MyoD are required for progenitor cells to become myoblasts, while Myf5, MyoD, and myogenin coordinate the differentiation of myoblasts into myocytes having an elongated shape. Myocytes fuse with neighboring cells to form multinucleated myotubes. The terminal stage of differentiation is mediated by the activation of MRF4 and genes responsible for muscle fiber (myofiber) architecture and functionality, such as myosin heavy chain (MHC). It has been reported that myogenesis is mediated by several signaling pathways, including the PI3K/AKT/p70S6K pathway (Sarker and Lee 2004), Raf/MEK/ERK pathway (Gredinger et al. 1998; Weyman and Wolfman 1998; Rommel et al. 1999), and PKA-dependent signaling pathway (Du et al. 2008). The PI3K pathway is crucial for myogenesis, while the PKA signaling pathway inhibits myogenic differentiation.

The β_2_-adrenergic receptor (β_2_-AR, ADRB2) is a class of G-protein-coupled receptors that is activated by the endogenous agonists’ catecholamines (Lynch and Ryall 2008). Traditionally, this receptor is known to interact exclusively with stimulatory Gα (G_s_) protein, which activates adenylyl cyclase, catalyzing the formation of cyclic adenosine monophosphate (cAMP) followed by the activation of protein kinase A (PKA). Furthermore, the stimulation of cAMP formation and PKA activation by β_2_-AR is also known to inhibit the Ras/ERK pathway via Raf-1 kinase inactivation and Mek-1 kinase inhibition (Carie and Sebti 2007).

β_2_-AR plays several roles in different muscle cell types. It enhances smooth muscle relaxation in the GI tract, uterus, and bronchi. For that reason, β_2_-agonists are commonly used for treating asthma and chronic obstructive pulmonary disease (Billington et al. 2017). In cardiac muscle cells, the activation of β_2_-AR causes inotropic stimulation and its agonists can be used as drugs for chronic heart failure (Ho et al. 2010). In addition, β_2_-AR is also found in skeletal muscle cells and the administration of β_2_-agonists is known to cause gains in skeletal muscle mass in mice and rats (Yang and McElligott 1989; Hinkle et al. 2002; Conte et al. 2012). The hypertrophy and anti-atrophy effects of β_2_-agonists are caused by the increase in muscle protein synthesis as well as a decrease in muscle protein degradation. However, the effects of β_2_-AR activation on skeletal muscle differentiation have yet to be fully elucidated.

We report here that the formoterol-mediated stimulation of β_2_-AR inhibited myoblast differentiation. We also found that formoterol specifically blocked the IGF-I-induced phosphorylation of AKT in myoblast differentiation, strongly suggesting that β_2_-AR signaling selectively inhibits IGF-mediated myogenesis by inhibiting the PI3K–AKT pathway.

## 2. Materials and methods

### 2.1. Antibodies and reagents

Anti-MHC antibody was obtained from the Developmental Studies Hybridoma Bank. Antibodies against GAPDH, phosphor-AKT (Ser-473), AKT, phosphor-ERK1/2 (Thr202/Tyr204) and ERK1/2 were purchased from Cell Signaling Technology, Inc. (Danvers, MA, USA). Horseradish peroxidase conjugated anti-mouse secondary antibodies were obtained from GE Healthcare Life Sciences (Little Chalfont, UK). Horseradish peroxidase conjugated anti-rabbit secondary antibodies were obtained from Santa Cruz Biotechnology (Dallas, TX, USA). Alexa Fluor 488 goat anti-mouse antibody and Hoechst 33342 were from Molecular Probes (Eugene, OR, USA). Formoterol hemifumarate and ICI-118,511 were from Bio-Techne Corporation (Minneapolis, MN, USA). Terbutaline hemisulfate was obtained from Sigma-Aldrich (St. Louis, MO, USA). All other reagents were from Sigma-Aldrich (St. Louis, MO, USA).

### 2.2. Cell culture and differentiation

Rat L6 myoblasts were obtained from ATCC (Manassas, VA, USA) and cultured in DMEM supplemented with 10% FBS, 4 mM L-glutamine, 100 unit/mL penicillin and 100 μg/mL streptomycin. For myogenic differentiation, L6 myoblasts at 90% confluence were transferred to differentiation medium, DMEM supplemented with 2% FBS, 4 mM L-glutamine, 100 unit/mL penicillin and 100 μg/mL streptomycin. The medium was replaced with fresh differentiation medium every two days. To examine the dose-dependent effect of formoterol on L6 myoblasts differentiation, L6 cells at 90% confluence were differentiated in the presence of either DMSO or the indicated concentration of formoterol for 72 h. For time-dependent treatment, L6 myoblasts at 90% confluence were differentiated in the presence of either DMSO or formoterol (100 nM) for the indicated times. To investigate the effect of LY294002 or PD98059 on myogenic differentiation, L6 myoblasts or myocytes were cultured in differentiation medium with or without formoterol (100 nM) in the presence of LY294002 (50 μM) or PD98059 (50 μM) for 72 h. For the stimulation of L6 myoblasts by growth factors, cells were starved with serum withdrawal in the presence or absence of formoterol (100 nM) for 2 h and then stimulated with insulin (100 ng/mL), IGF-1 (20 ng/mL) or EGF (20 μg/mL) for 5 min. Whole cell lysates were subject to western analysis.

### 2.3. Immunofluorescence

L6 cells were grown on acid-washed 12-mm glass coverslips and differentiated with or without formoterol (100 nM). After fixed with 3.7% formaldehyde, cells were subsequently incubated with the anti-MHC antibody, Alexa Fluor 488-conjugated anti-mouse antibody, Hoechst 33342, and imaged with a confocal microscope (Carl Zeiss, Jena, Germany) as previously described (Yi et al. 2018).

### 2.4. Western blot analysis

L6 myoblasts were cultured at 90% confluence and differentiated. Myoblasts and differentiated myotubes were homogenized with lysis buffer (25 mM Tris, pH 7.9, 150 mM NaCl, 0.5% NP-40, 1 mM EDTA, 5% glycereol, protease inhibitors). Protein concentration of cell lysates was determined by the Protein Assay Kit (iNtRON biotechnology, Korea), an equal amount of cells lysates was separated by SDS-PAGE and transferred to PVDF membranes (GE Healthcare, Germany). The membranes were incubated with specific antibodies against MHC, p-AKT, AKT, p-ERK, ERK, and GAPDH. The blots were then incubated with horseradish peroxidase conjugated anti-mouse secondary antibodies or horseradish peroxidase conjugated anti-rabbit secondary antibodies. Bands were detected by the ECL detection system (GE Healthcare, Germany) and band intensities were quantified by ImageJ software (Yi, Hwang, Oh, Kim, Ryu, et al. 2018).

### 2.5. Statistical analysis

Results are obtained from three independent experiments. Data are expressed as means ± standard deviation of the mean (SD). The results were statistically analyzed by one-way ANOVA method. The results were analyzed by one-way ANOVA and the significance was examined with Fischer’s Protected LSD post-hoc test. A *P* values < 0.05 was considered statistically significant.

## 3. Results

### 3.1. Formoterol inhibits L6 myoblast differentiation

**Figure 1.**
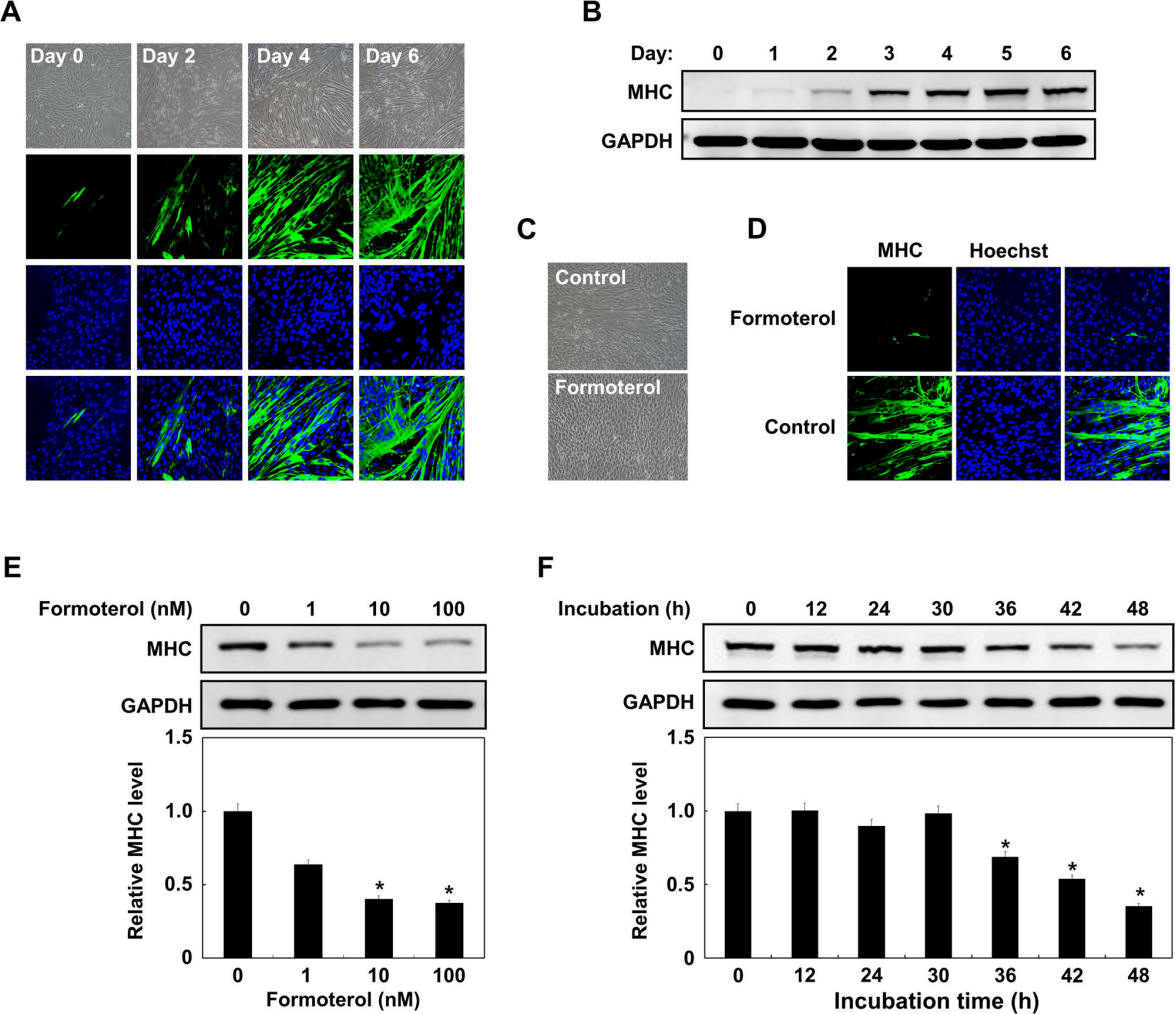
Inhibition of L6 myoblast differentiation by β_2_-adrenergic receptor agonist, formoterol. (A) L6 myoblasts were induced to differentiate for the indicated time. The differentiated myocytes were identified by immunostaining of myosin heavy chain (MHC) (green) as a differentiation marker. Nuclei (blue) were stained with Hoechst 33342. (B) The differentiated myocytes were lysed for immunoblotting with either anti-MHC antibody or anti-GAPDH antibody. (C) The myoblasts (90% confluence) were differentiated in the presence of DMSO or β2-adrenergic receptor agonist, formoterol (100 nM), for 72 h. The morphological changes of myoblasts upon formoterol treatment were observed by phase contrast microscopy. (D) The cells treated with formoterol as in C were fixed and analyzed for the expression of MHC protein and the formation of multinucleated muscle cells by immunostaining with anti-MHC antibodies (green) and Hoechst dye (blue). (E) L6 myoblasts were differentiated with formoterol (1–100 nM) for 72 h. Then, the cells were lysed and immunoblotted with anti-MHC antibody, band intensities were quantified using ImageJ software, and the relative intensities of three independent experiments are presented as mean ± SD. Values from cells treated with DMSO are set to 1. *p < 0.05 relative to control cells treated with DMSO. (F) L6 myoblasts were differentiated in the presence of formoterol (100 nM) for the indicated times, and the cells were lysed and immunoblotted with anti-MHC antibody. Band intensities were quantified as in (E). Results are obtained from three independent experiments. Values from cells treated with DMSO are set to 1. *Bars*, the mean result ± SD. *p < 0.05 relative to control cells treated with DMSO.

A recent study demonstrated that β_2_-AR mRNA expression is induced during muscle cell differentiation (Wannenes et al. 2012). In addition, several in vivo studies have shown that formoterol treatment improves skeletal muscle anabolism and hypertrophy (Yang and McElligott 1989; Conte et al. 2012). The aforementioned studies prompted us to investigate the effect of formoterol on the differentiation of myoblasts. To do this, we first established the myoblast differentiation of L6 rat skeletal myoblasts by examining the expression of MHC as a marker of differentiation. As shown in Figure 1A, nearly all 2-d myoblast-induced cells were mononuclear pre-myocytes that fuse to form large multinuclear myocytes (myotube formation) by 6 days. Immunostaining and western blot analyses showed that MHC proteins were increased during myoblast differentiation (Figure 1A and B). To examine the effect of formoterol on the differentiation of L6 myoblasts, L6 myoblast cells cultured in differentiation medium were treated with formoterol for 3 days. Unexpectedly, myotube formation was completely blocked by formoterol treatment (Figure 1C and 1D). We further investigated the dose and time dependence of formoterol-mediated inhibition of myotube formation. L6 myoblast cells were treated with an increasing amount of formoterol up to 100 nM for 3 days. Then, MHC expression was monitored by western blotting with the anti-MHC antibody. As shown in Figure 1E, formoterol inhibited myotube formation in a dose-dependent manner, with maximal inhibition at 10 nM. To analyze the time-dependent inhibitory effect of formoterol, cells were treated with differentiation medium containing 100 nM formoterol for the indicated time (Figure 1F; 12, 24, 30, 36, 42, and 48 h). Next, the cells were transferred to normal differentiation medium and then their expression level of MHC was analyzed by western blotting. Short-term (up to 30 h) treatment with formoterol had a less inhibitory effect on MHC expression. Interestingly, however, MHC expression was sharply blocked with longer formoterol treatment (36–48 h).

### 3.2. β_2_-AR is linked to L6 myoblast differentiation

**Figure 2.**
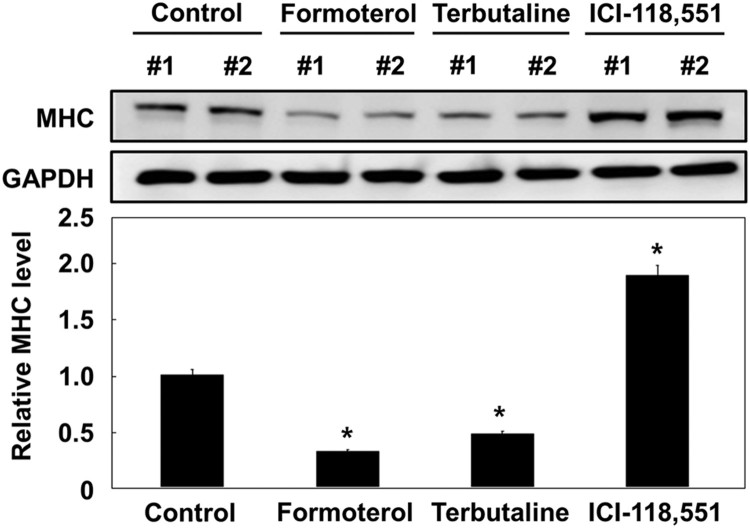
Effects of other β_2_-AR agonists and antagonist on L6 myoblast differentiation. L6 myoblasts were differentiated under control conditions (10% DMSO) or in the presence of formoterol (100 nM), terbutaline (3 μM), ICI-118,551 (100 nM), or formoterol (100 nM) plus ICI-118,551 (100 nM) for 72 h. The treated cells were lysed and immunoblotted with anti-MHC antibody. Band intensities were quantified and normalized to GAPDH control. Results are obtained from three independent experiments. Values from cells treated with DMSO are set to 1. *Bars*, the mean result ± SD. *p < 0.05 relative to control cells treated with DMSO.

We next investigated whether formoterol inhibited myoblast formation through the β_2_-adrenergic receptor signaling pathway, but not other pathways. To this end, we employed two small compounds: one is a short-acting β_2_-adrenergic receptor agonist (terbutaline), and the other is a selective β_2_-adrenergic receptor antagonist (ICI-118,551). As shown in Figure 2, both formoterol (long-acting) and terbutaline (short-acting) similarly decreased MHC protein expression. However, ICI-118,551 treatment increased MHC protein expression. These results demonstrate that the inhibition of L6 myogenesis by formoterol is specifically mediated through the β_2_-adrenergic receptor, not through other receptors.

### 3.3. Formoterol inhibits L6 myoblast differentiation via the PI3K–AKT signaling pathway

**Figure 3.**
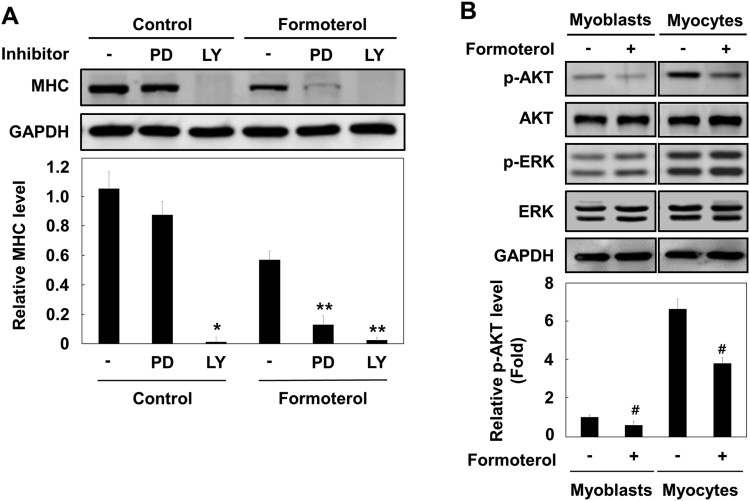
Effect of formoterol on PI3K–AKT signaling pathway. (A) L6 myoblasts were differentiated in the presence of PD98059 (50 μM) or LY294002 (20 μM) with and without formoterol (100 nM) for 72 h. The cells were lysed and immunoblotted with anti-MHC. Band intensities were quantified and normalized to GAPDH control. Results are obtained from three independent experiments. Values from cells treated with DMSO are set to 1. *Bars*, the mean result ± SD. *p < 0.05 relative to control cells treated with only DMSO, **p < 0.05 relative to cells treated with only formoterol. (B) L6 myoblasts or L6 myocytes (serum starvation for 48 h) were incubated with either DMSO or formoterol for 5 min. The cells were lysed and immunoblotted with antibodies against p-Akt, Akt, p-ERK, and ERK. Results are obtained from three independent experiments. Values from cells treated with only DMSO are set to 1. *Bars*, the mean result ± SD. ^#^*p *< 0.05 relative to L6 cells treated with only DMSO.

Many intracellular signaling pathways are involved in myogenesis, including p38 MAPK, ERK1/2, and PI3K/AKT. The p38 and ERK1/2 pathways differentially activate myogenic differentiation at multiple steps (Wu et al. 2000). In addition, PI3K–AKT signaling is essential for myogenic differentiation (Bodine et al. 2001). As formoterol has an inhibitory effect on L6 myogenesis, we next determined which signaling pathway is affected by formoterol treatment. To do this, we used selective pathway inhibitors: LY294002, a PI3K/AKT pathway inhibitor, and PD98059, a Ras/ERK pathway inhibitor. L6 myoblast cells were differentiated and treated with PD98059 or LY294002 in the absence or presence of formoterol, and MHC protein expression was analyzed with MHC antibody. As shown in Figure 3A, the treatment of LY294002 with or without formoterol showed the strong suppression of MHC expression, implying that the PI3K/AKT pathway plays a critical role in L6 cell differentiation. While PD98059 alone had litle effect on MHC expression, PD98059 plus formoterol apparently decreased MHC expression. To further discriminate whether formoterol blocks either PI3K, or MAPK or both signaling pathway, we examined the activation of AKT and ERK proteins by formoterol in both myoblasts (early stage of myogenic differentiation) and myocytes (middle stage of myogenic differentiation). We found that both AKT and ERK pathways are increasingly activated during the myogenic differentiation (Figure 3B, compare myoblast versus myocyte). Formoterol significantly inhibited the phosphorylation of AKT in both myoblast and myocytes. In contrast, the phosphorylation of the ERK protein was unaffected (Figure 3B). These results suggest that formoterol inhibits L6 muscle cell differentiation mainly through the PI3K/AKT pathway.

### 3.4. Formoterol inhibits IGF- I-mediated activation of AKT

**Figure 4.**
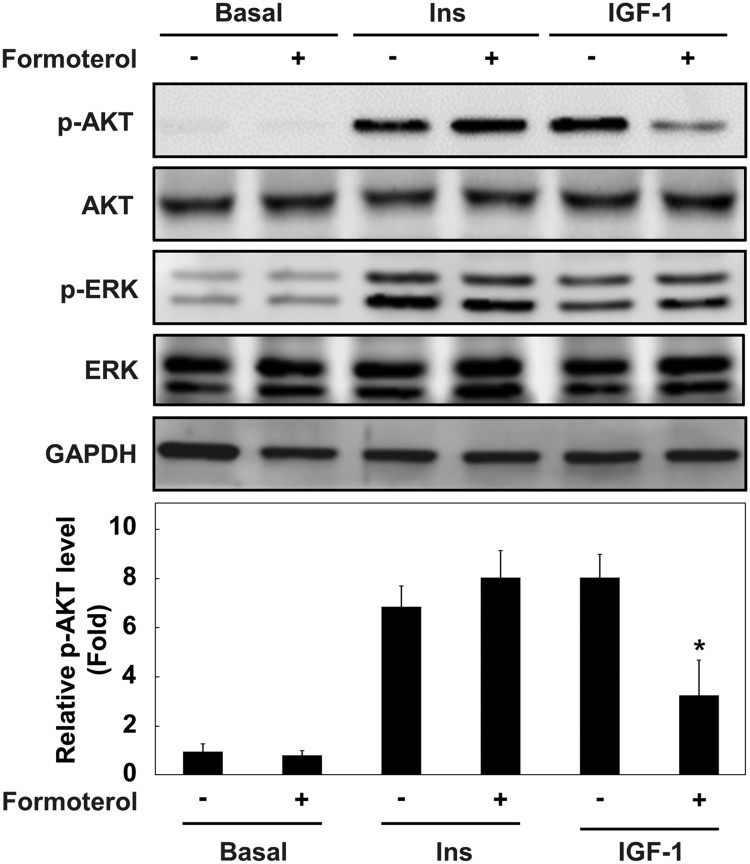
Effects of formoterol on the signaling pathway of insulin and IGF-I. The cell cycle of L6 myoblasts was arrested by serum withdrawal in the presence or absence of formoterol (100 nM) for 2 h, and then stimulation with either insulin (100 ng/ml) or IGF-1 (10 ng/ml) was applied for 5 min. The cells were lysed and immunoblotted with antibody against p-Akt, Akt, p-ERK, and ERK. Band intensities were quantified and normalized to GAPDH control. Results are obtained from three independent experiments. Values from basal cells treated are set to 1. *Bars*, the mean result ± SD. *p < 0.05 relative to L6 myoblasts stimulated with only IGF-I.

Growth factors, such as insulin and IGF-I, are known to promote myogenic differentiation by activating the PI3K–AKT signaling pathway (Conejo et al. 2001; Sarker and Lee 2004). To further determine whether insulin and/or IGF-I-mediated AKT activation is regulated by formoterol, L6 myoblasts were serum-starved and stimulated with insulin and IGF-I in the presence or absence of formoterol. As shown in Figure 4, insulin and IGF-I stimulated AKT activation. Formoterol treatment selectively inhibited AKT activation by IGF-I, but not by insulin. By contrast, ERK activation by insulin and IGF-I was unaffected. These results suggest that β_2_-adrenergic receptor activation by formoterol differentially regulates the AKT signaling pathway by insulin and IGF-I, although the molecular mechanism underlying the differential regulation is currently unknown.

## 4. Discussion

The differentiation of skeletal muscle is a highly complex process involving the formation of multinucleated muscle fibers. Although a growing body of evidence suggests that formoterol, a β_2_- agonist, may have a profound effect on skeletal muscle hypertrophy (Yang and McElligott 1989; Conte et al. 2012), the molecular mechanisms involved are unclear. In the present study, we focused our investigation on the roles of β_2_-AR stimulation in myogenic differentiation. Unexpectedly, our in vitro pharmacological approaches using two agonists of β_2_-AR revealed that the activation of β_2_-AR significantly inhibits the expression levels of MHC protein. Furthermore, formoterol sufficiently inhibited myotube formation using several biochemical methods as well as morphological changes in L6 rat muscle cells. These findings strongly indicate that β2AR regulates myoblast differentiation in L6 rat muscle cells. Our result is somewhat inconsistent with a previous study showing that β_2_-agonist treatment did not affect skeletal muscle differentiation (Wannenes et al. 2012). Wannenes et al. mainly investigated the effect of β_2_-agonists on mRNA and protein expression regarding hypertrophy and atrophy in C2C12 mouse muscle cells. Such inconsistency may be due to the difference in cell lines and differentiation conditions. Further studies will be needed to resolve these discrepancies.

**Figure 5.**
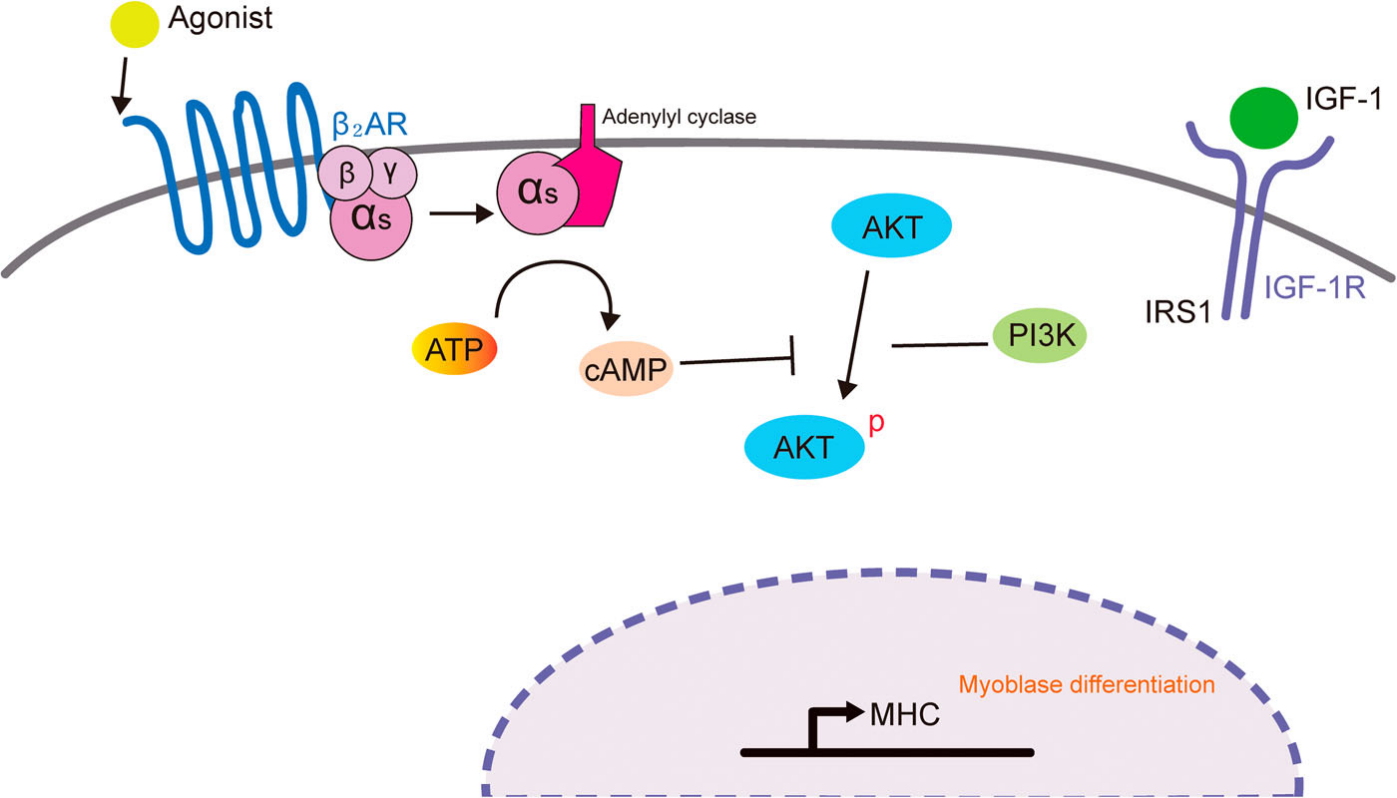
Proposed model of the mechanism by which β_2_-AR activation inhibits myogenic differentiation by blocking the PI3K–AKT pathway. IGF-I activates IGF-IR to stimulate myogenic differentiation through the PI3K–AKT signaling pathway. Upon β_2_-agonist (e.g. formoterol) binding to β_2_-adrenoreceptor, adenylyl cyclase is activated, resulting in the accumulation of cAMP and activation of PKA. Cyclic AMP and activated PKA may block the PI3K–AKT signaling pathway, which will lead to the inhibition of myogenic differentiation.

Several signaling pathways such as p38 MAPK, ERK1/2, and PI3K/AKT are known to activate myogenic differentiation at multiple steps (Gredinger et al. 1998; Weyman and Wolfman 1998; Rommel et al. 1999; Wu et al. 2000; Bodine et al. 2001). The use of pharmacological inhibitors (PD98059 and LY294002) against these pathways revealed that the PI3K–AKT pathway is crucial for myogenic differentiation in L6 muscle cells. Furthermore, the activation of β_2_-AR with formoterol specifically blocked AKT phosphorylation, but not ERK phosphorylation. Considering the previous report describing that cyclic AMP (cAMP) and cAMP-dependent protein kinase (PKA) inhibit the PI3K–AKT pathway (Kim et al. 2001), it is possible that formoterol-mediated activation of PKA signaling suppresses AKT phosphorylation (Figure 5).

An intriguing finding of our study is that formoterol preferentially inhibits AKT phosphorylation induced by IGF-I, but not by insulin. Although both growth factors are required for myoblast differentiation and share downstream effectors to transmit signals, recent studies have demonstrated that insulin and IGF-I mediate distinct cellular and physiological functions (Palsgaard et al. 2009; Cai et al. 2017). Palsgaard et al. showed that IGF-I is a more potent metabolic regulator than insulin in skeletal muscle cells. Defining the molecular basis of the differential regulation on AKT activity is beyond the scope of this study, but will be of interest to determine how formoterol differentially controls AKT phosphorylation in myoblast cells in response to insulin or IGF-I stimulation.
